# Mechanosensitive ion channels in cell migration

**DOI:** 10.1016/j.cdev.2021.203683

**Published:** 2021-06

**Authors:** Brenda Canales Coutiño, Roberto Mayor

**Affiliations:** Department of Cell and Developmental Biology, University College London, Gower Street, London WC1E 6BT, UK

**Keywords:** Cell migration, Mechanics, Mechanotransduction, Calcium signalling, Ion channels

## Abstract

Cellular processes are initiated and regulated by different stimuli, including mechanical forces. Cell membrane mechanosensors represent the first step towards the conversion of mechanical stimuli to a biochemical or electrical response. Mechanosensitive (MS) ion channels form a growing family of ion gating channels that respond to direct physical force or plasma membrane deformations. A number of calcium (Ca^2+^) permeable MS channels are known to regulate the initiation, direction, and persistence of cell migration during development and tumour progression. While the evidence that links individual MS ion channels to cell migration is growing, a unified analysis of the molecular mechanisms regulated downstream of MS ion channel activation is lacking. In this review, we describe the MS ion channel families known to regulate cell migration. We discuss the molecular mechanisms that act downstream of MS ion channels with an emphasis on Ca^2+^ mediated processes. Finally, we propose the future directions and impact of MS ion channel activity in the field of cell migration.

## Highlights

• Cellular processes are initiated and regulated by different stimuli, including mechanical forces.
• Mechanosensitive (MS) ion channels form a family of channels that respond to physical force or plasma membrane deformations.
• A number of MS channels are known to regulate the initiation, direction and persistence of cell migration.

## 1 Introduction

Living organisms are exposed to a wide array of mechanical cues, from universal forces like gravity to microscopically localized stimuli such as fluid shear stress in blood vessels (Lu and Kassab, 2011), compression by neighbouring tissues (Barriga et al., 2018; Kim et al., 2017), or extracellular matrix stiffness (Chaudhuri et al., 2020). Evolutionarily, living organisms have adapted to the forces surrounding them via mechanosensitive proteins within the cell (Cox et al., 2018; Fritzsch et al., 2007). Mechanosensitive proteins are essential to detect a mechanical cue and convert a mechanical force to a biochemical cascade, through the process known as mechanotransduction. An important family of mechanosensors are mechanosensitive (MS) ion channels, which are pore-forming protein structures localized in the cell plasma membrane and the membrane of certain organelles, i.e. the endoplasmic reticulum, endosomes, and lysosomes (Dong et al., 2010; Dong et al., 2008; Santana et al., 2019). MS ion channels are activated by mechanical forces thus allowing ion transport. The majority of MS ion channels have high specificity for calcium ions (Ca^2+^); therefore, they are often referred to as stretch-activated Ca^2+^ channels. MS ion channels are an important link between mechanical stimuli and Ca^2+^ mediated signalling and have been described to regulate the initiation, persistence, and directionality of cell migration. This review aims to provide the reader with a unified understanding of the molecular mechanisms by which MS ion channels regulate cell migration. We start with a description of the different MS ion channels that have been linked to cell migration. We then present a detailed analysis of the pathways and mechanisms of action downstream of MS ion channels during mechanotransduction in cell migration, with emphasis on Ca^2+^ mediated signalling. Finally, we outline the emerging MS ion channels and discuss the key future directions.

## 2 The mechanics of cell migration

Cell migration is the process where an individual or a group of cells (collective migration) move from one location to another. Cell migration is an essential process during embryonic development, as cells migrate to accommodate tissue rearrangement and can travel long distances to the tissues where they will eventually differentiate into specialized cell types. Some widely studied examples include *Drosophila* border cells (Prasad et al., 2015), zebrafish lateral line primordium (Olson and Nechiporuk, 2018), convergent extension during gastrulation (Tada and Heisenberg, 2012), neural crest cells in *Xenopus*, chick and zebrafish (Shellard and Mayor, 2019). Additionally, cells are highly migratory in certain diseases, such as the invasion of malignant cells during cancer metastasis (Stuelten et al., 2018), and cells of the immune system that travel to a site of bacterial or viral infection (Yamada and Sixt, 2019). Cell migration is also one of the mechanisms that cells adopt during wound closure after tissue damage (Xiao et al., 2019).

**Fig. 1:**
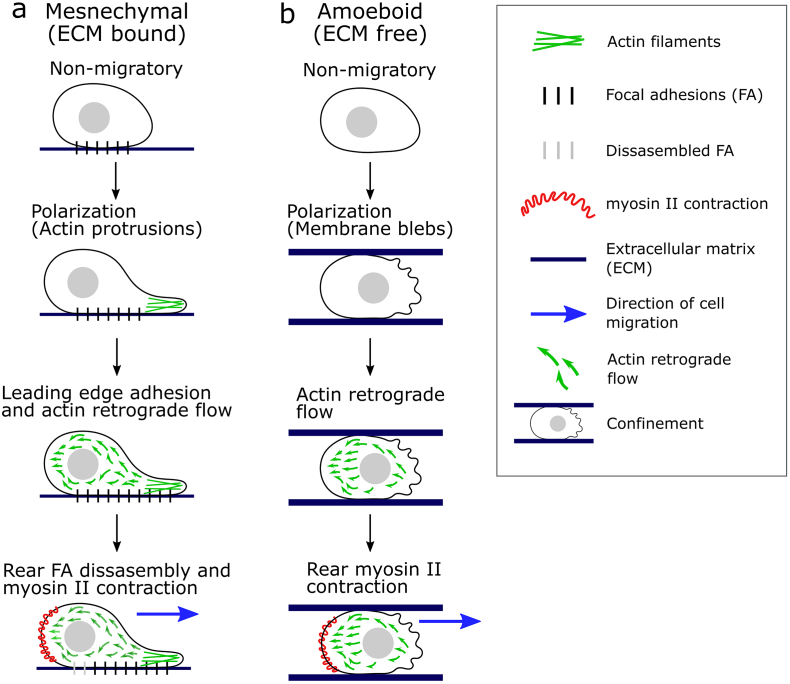
Basic steps of cell migration. **(a)** Mesenchymal cell migration. Cells are attached to the extracellular matrix (ECM) via integrins and focal adhesions (FA). Actin polymerization at the leading edge extends filamentous actin (F-actin) protrusions inducing a front-rear polarization. New FA adhesions attach the protrusions to the ECM followed by F-actin rearward movement, known as actin retrograde flow. Disassembly of rear FA and myosin II contraction at the back of cell generate the pushing force to move the cell forward. **(b)** Amoeboid cell migration. Cells do not form adhesions with the ECM or other cells. Under confinement, amoeboid cells form membrane blebs, also known as pseudopodia, inducing a front-rear polarization. Actin retrograde flow is initiated by mechanical forces, such as confinement. Myosin II contraction at the back of cell generates the pushing force to move the cell forward.

The initiation of cell migration requires a step of front-rear cell polarization. In mesenchymal cell migration, a leading front extends actin-based protrusions, these connect to the extracellular matrix (ECM) via integrins that provide the traction necessary for cell movement (Fig. 1a). Intracellularly, integrins connect to the actin cytoskeleton via focal adhesion (FA) proteins. The integrin-FA complex acts as a mechanosensor, an increase in the matrix stiffness promotes the activation and clustering of integrins and FAs (Wei et al., 2008; Friedland et al., 2009), which feedback to the actin filaments inducing re-arrangement of the cytoskeleton structure, thereby affecting cell migration (Matthews et al., 2006). In addition to traction forces, actin retrograde flow accompanied by myosin II-induced contraction at the rear of cells generates pushing forces to propel the cell forward (Fig. 1a). Actomyosin contraction and actin retrograde flow are also essential for integrin-free migration (also known as amoeboid migration), where cells are not attached to the ECM by FAs (Yolland et al., 2019). In amoeboid migration, actin retrograde flow is induced by strong mechanical stimuli, such as confinement, instead of integrin/FA mechanotransduction (Liu et al., 2015) and membrane blebbing is the main mechanism for cell polarization, instead of actin-based protrusions (Fig. 1b). Amoeboid migration is the mechanism for the cells of the immune system (Lämmermann et al., 2008) and in several cancers (Khoo et al., 2019; Canales Coutiño et al., 2020). Additionally, ameboid migration is the most efficient mechanism under extreme confinement, i.e. cells travelling in the smallest capillary vessels (approximately 4 μm in size) (Au et al., 2016).

Cells undergoing migration are especially susceptible to many different mechanical stimuli, as cells under movement are constantly encountering different tissues and mechanical conditions. MS ion channels localized across the plasma membrane can be activated by a myriad of stimuli during the journey of a migrating cell, including compression by nearby tissues (Barriga et al., 2018; Kim et al., 2017), stiffness of ECM (Chaudhuri et al., 2020), and shear stress for cells migrating in blood vessels (Lu and Kassab, 2011). Furthermore, self-generated forces of a migrating cell, due to actomyosin contraction, have been shown to activate MS ion channels (Koser et al., 2016). Overall, the dynamic nature of the cell migration process and the microenvironment surrounding the migrating cells generate mechanical cues that can activate MS ion channels and induce a change in local Ca^2+^ levels. Localized regulation of Ca^2+^ levels, as observed in Ca^2+^ flickers, directly affects cell migration via Ca^2+^ dependent proteins (Wei et al., 2009). As Ca^2+^ gating channels, MS ion channels from the transient potential receptor (TRP) and Piezo families have been shown to play an important role in the regulation of cell migration.

## 3 TRP superfamily and Piezo MS ion channels

MS calcium channels were first described in the early 1980s, where single ion currents were detected after stimulation of chick embryos with a patch-clamp (Guharay and Sachs, 1984). For decades, MS ion channels were thought to act exclusively as propagators of electric signals to the central nervous system. There are now several biological roles linked to MS ion channels in non-neuronal cells, including pathways that affect several aspects of cell migration. Two families of MS ion channels are currently known to regulate cell migration: transient receptor potential (TRP) and Piezo channels. The following sections present an overview of these MS ion channels and how they have been linked to mechanotransduction.

### 3.1 TRP channels

**Fig. 2:**
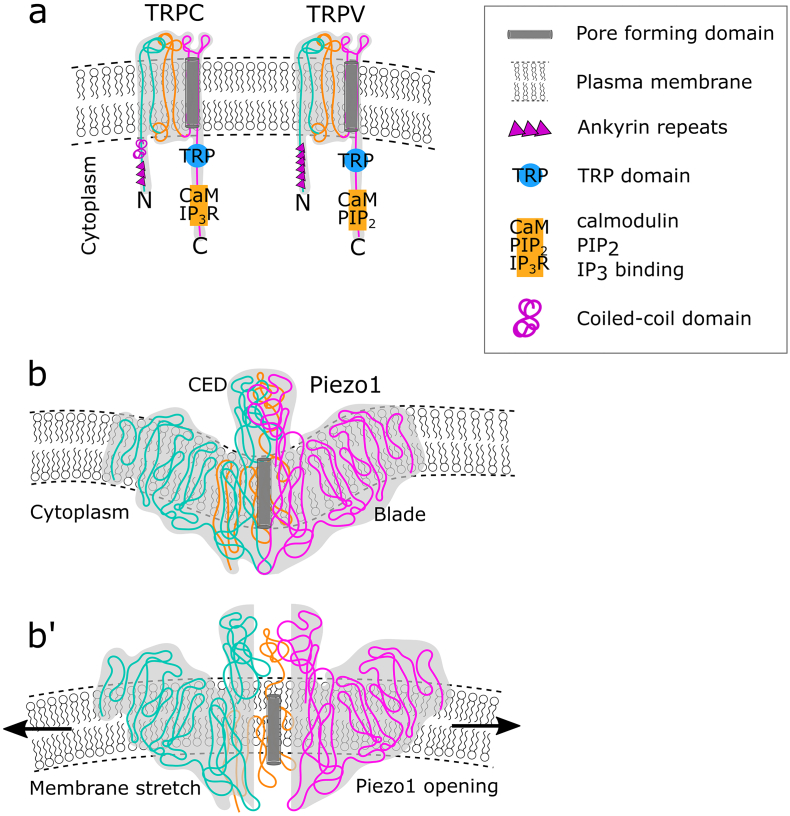
MS ion channel families involved in cell migration. **(a)** Transient receptor potential channels (TRP). TRP channels form 6 transmembrane (TM) domains. TM 1-2 are represented in cyan, TM 3-4 in orange and TM 5-6 in magenta. The pore forming domain is formed between TM5 and TM6. Each subfamily of TRP channels contains unique domains in the cytoplasmic N- and C- termini. TRPC channels have three ankyrin repeats and a coiled-coil domain in the N-terminus. A TRP domain, which has gating functions, a calmodulin and IP3R binding domains are localized in the C-terminus. TRPV channels have six ankyrin repeats in the N-terminus. A TRP domain, a calmodulin and PIP2 binding domains are localized in the C-terminus. **(b–b′)** Piezo1 channels. **(b)** Each Piezo1 channel has at least 26 TM regions and up to 40 TM domains. The TM domains form three defined structures, known as blades. Each blade is colour coded in cyan, orange and magenta for easier representation. The carboy-terminal extracellular domain (CED) is located directly on top of the pore forming domain and is important for ion selectivity (Zhao et al., 2016). **(b′)** Due to its large size, a Piezo1 channel induces a small curvature to the plasma membrane, when force is applied the plasma membrane is stretched, thereby opening the Piezo1 channel.

TRP is a superfamily of ion channels. There is a total of 28 TRP mammalian channels, which are categorized into 6 different subfamilies based on sequence homology, the subfamilies are TRPC (canonical), TRPV (vanilloid), TRPM (melastatin), TRPA (ankyrin), TRPP (polycystin) and TRPML (mucolipin). Each TRP channel consists of 6 transmembrane (TM) domains, with the pore-forming domain localized between TM5 and TM6 (Fig. 2a). TRP channels are a very diverse group, although they are all permeable to cations, their pattern of expression and mode of activation varies greatly, even within members of a specific subfamily. For example, members of the TRPV subfamily can be activated by temperature, chemical stimuli, pH changes, low cation levels (store-operated) and mechanical stress; meaning that not all TRPV channels are mechanosensitive. Additionally, some TRP channels are expressed in a tissue-specific manner, the majority of which are exclusively localized in sensory neurons or mechanically specialized cells such as sensory hair cells of the inner ear. Here, we focus on describing the TRP channels that are both mechanosensitive and are known to have a role in cell migration, these include members of the TRPC and TRPV subfamilies. Mechanosensitive TRP channels that have only been studied in non-migratory cells are beyond the scope of this paper. We direct the reader to these reviews that describe the mechanosensitive aspect of the TRP superfamily more extensively (Liu and Montell, 2015; O'Neil and Heller, 2005).

#### 3.1.1 Transient receptor potential canonical

TRPC channels are the founding members of the TRP superfamily, first discovered in the retina of *Drosophila* as photoreceptors (Cosens and Manning, 1969). Mammalian TRPC channels were identified based on homology to the *Drosophila* channels (Wes et al., 1995). Mammalian TRPC channels are expressed in a wide variety of tissues, unlike the *Drosophila* orthologs that are restricted to the retina cells. There are seven TRPC channels in mammalian cells (TRPC1-7). The protein structure, determined by single-particle cryogenic electron microscopy (cryo-EM), has been obtained for TRPC3-6 (Duan et al., 2019; Tang et al., 2018), which has offered insights into the gating mechanism of TRPC channels. Structurally, all TRPC channels have four ankyrin repeats next to the cytoplasmic N-terminal, which mediate protein-protein interaction (Fig. 2a). Multiple coiled-coil domains are localized upstream of the TM1 domain. A TRP domain, which has channel gating functions, is in the cytoplasmic region next to the TM6 domain. There are calmodulin and IP3R binding domains in the C-terminal region (Fig. 2a), TRPC channels can be activated by IP3 and inactivated by calmodulin when intracellular Ca^2+^ levels are high, the store operated activation of these channels is regulated by these domains. TRPC channels can also be activated downstream from mechanosensitive G-protein coupled receptors that signal through phospholipase C (PLC). TRPC mechanical activation can be mediated directly by membrane deformations or indirectly and dependant on mechanosensitive G-protein coupled receptors. Within the TRPC family TRPC1, TRPC5 and TRPC6 are known to be activated by mechanical stimuli (Box 1).

Box 1 **TRPC1** was first identified as mechanosensitive in *Xenopus* oocytes. Expression of an antisense morpholino for TRPC1 is sufficient to lower the ion currents recorded via patch-clamp after applying pressure with a Piezoelectric transducer. Mechanosensitivity of this channel can be rescued by the expression of human TRPC1 (Maroto et al., 2005). Since then, TRPC1 has been characterized as an MS ion channel in root ganglion neurons (Staaf et al., 2009), mice myocardial tissue (Seth et al., 2009), axons (Kerstein et al., 2013), glioblastoma cells (Huang et al., 2015), tumour-associated pancreatic stellate cells (Fels et al., 2016) and bronchial epithelial cells (Li et al., 2019; Wang et al., 2020). Additionally, Mammalian TRP proteins can be found as heterocomplexes. Heterochannels formed by the binding of TRPC1 to TRPC5 leads to the generation of novel channels with biophysical properties distinct from those of TRPC1 and TRPC5 individual channels (Strübing et al., 2001). TRPC1 can also form complexes with TRPP2, TRPV4 and TRPC4 (Maroto et al., 2005; Chen and Barritt, 2003). The mechanical sensibility of TRP heterocomplexes formed by mechanosensitive and non-mechanosensitive TRP proteins, i.e. TRPC1 and TRPC4, is yet to be studied and might provide a better understanding of the properties and mechanisms for TRP channel regulation. Heterochannels formed between TRP proteins confer an additional layer of complexity to the role of TRP channels in mechanotransduction.

**TRPC5** is the most recent TRPC channel linked to mechanosenstivity. TRPC5 channels can be activated by membrane stretching or osmotic pressure in HEK293 cells (Gomis et al., 2008; Shen et al., 2015). The TRPC5 channel is the only TRP channel specifically localized in the apical membrane of airway epithelial cells in rodents and acts as an important mechanotransductor of osmotic pressure (Lembrechts et al., 2012). TRPC5 channels can also regulate vascular tone (Liang et al., 2019) and angiogenesis in mice (Zhu et al., 2019). TRPC5 channels, like the other members of the subfamily, can be activated in a PLC dependent manner. However, unlike other TRPC channels, TRPC5 is insensitive to IP3 (Venkatachalam et al., 2003). These small differences in channel structure and response to stimuli are an opportunity for further research to understand the specific biological functions related to each MS ion channel.

**TRPC6** has been identified as mechanosensitive in myocyte muscular cells in response to intravascular pressure in arteries (Welsh et al., 2002). The function of TRPC6 is controlled by both tension and curvature of the surrounding lipid bilayer leading to an increase of Ca^2+^ influx and elevated Na^+^ levels (Dyachenko et al., 2009). Unlike other TRPC channels, the mechanical activation of TRPC6 in smooth muscle cells was found to be independent of chemical stimuli, including the TRPC activator, PLC (Spassova et al., 2006). TRPC6 has only been identified as mechanosensitive in vascular smooth muscle cells although it is expressed in a wide variety of tissues. This raises the question of whether TRPC6 confers mechanosensitivity to other cell types. Additionally, TRPC6 channels can form heteromers with TRPC3 and TRPC7 (Lepage et al., 2006), thereby they have the potential to form a large number of unique ion channels.

**TRPV2** can be activated by cell swelling and membrane stretch in vascular smooth muscle cells, leading to elevated intracellular Ca^2+^ levels (Muraki et al., 2003). Expression of TRPV2 in hamster ovary K1 cells, which are otherwise non-mechanosensitive, confers response to membrane stress through the patch-clamp technique (Muraki et al., 2003). Additionally, TRPV2 acts as a mechanosensor in mice smooth muscle cells in response to osmotic stress (Iwata et al., 2009). TRPV2 is expressed in several cell types and is required for directional migration of macrophages (Link et al., 2010), prostate tumours (Monet et al., 2010), bladder cancer (Liu and Wang, 2013) and oesophageal squamous cell carcinoma (Kudou et al., 2019). Whether TRPV2 acts as a mechanosensor in these cells is yet to be studied and could provide a better understanding of the mechanism of action of TRPV2.

**TRPV4** is the most characterized TRP member in cell migration. TRPV4 is associated with the response to osmotic pressure (Liedtke, 2005; Mamenko et al., 2015) and shear stress (Zhang and Gutterman, 2011; Köhler and Hoyer, 2007). The mechanosensitive nature of TRPV4 was found to be dependent on the actin cytoskeleton directly interacting with ankyrin repeats located in the N-terminus of TRPV4. Deletion of the ankyrin repeat domain dramatically impairs TRPV4 mechanosensitivity by disrupting its association with the cytoskeleton, which possibly provides a mechanical link for gating (Liedtke et al., 2000). Additionally, TRPV4 high expression is linked to increased cell migration in endothelial cells (Fiorio Pla et al., 2012), pulmonary smooth muscle cells (Martin et al., 2012), breast cancer cells (Lee et al., 2016), glioblastoma (Yang et al., 2020; Ou-Yang et al., 2018).

Alt-text: Box 1

#### 3.1.2 Transient receptor potential vanilloid

The TRPV subfamily consists of six members TRPV1–6. TRPV1 was the first channel identified within this subfamily and is mainly activated by high temperatures. The other TRPV members were categorized based on sequence homology to TRPV1. The TRPV subfamily has been extensively studied due to the heat sensitivity of its founding member, however, several TRPV members were found to be irresponsive to temperature. TRPV channels are now known as the TRP family that is activated by the widest variety of stimuli, including mechanical stimuli such as stretch, changes in osmotic pressure and indirectly by PLC release from mechanosensitive G-protein coupled receptors.

TRPV proteins share high homology with TRPC channels over the region spanning the pore-forming domains TM5 and 6. The biggest structural difference compared to TRPC is that TRPV channels do not have multiple coil coiled domains (Fig. 2a). High-resolution structures have been acquired for TRPV1, 2 and 6 using cryo-EM X-ray crystallography (Gao et al., 2016; Liao et al., 2013; Huynh et al., 2016; Zubcevic et al., 2016; Saotome et al., 2016). A major leap in the understanding of TRPV1 response to different stimuli, reported by Gao et al., was the cryo-EM analysis of the TRPV1 structure performed while the channel was exposed to different pharmacological drugs, a peptide toxin and small vanilloid agonists. Two pore-forming gates support a dual gating mechanism, called upper and lower gate. These undergo substantial conformational changes associated with gating and were found to be key in TRPV1 response to different chemical stimuli (Cao et al., 2013). Although TRPV1 is not mechanosensitive, the discoveries of the different gating regulatory mechanisms can provide a clue to the regulation of mechanosensitive MS ion channels that also respond to chemical stimuli. Within the TRPV family, TRPV2 and TRPV4 are mechanoproteins, sensitive to hypotonic cell swelling, shear stress, and membrane stretch (Box 1).

### 3.2 Piezo channels

The Piezo family of channels is formed by two members, Piezo1 which is expressed in a wide variety of tissues, and Piezo2 that is expressed in sensory neurons that respond to touch (Coste et al., 2010). Unlike TRP channels, Piezo channels are exclusively activated by mechanical stimuli, this provides an advantage for mechanotransduction studies since the biological response can be directly attributed to a mechanical cue. Due to their direct mechanosensitivity, Piezo channels respond to mechanical cues within milliseconds after applying force (Nilius and Honoré, 2012), while signal after TRP channel mechanical activation can take up to 30 s (Berrout et al., 2012; Everaerts et al., 2010; Nilius et al., 2004).

High-resolution cryo-electron microscopy structure of the mouse Piezo1 (Saotome et al., 2018) depicts that its TM domains form four bundles; three TM bundles in a conformation resembling propeller blades and one extracellular bundle known as CED (C-terminal extracellular domain) (Fig. 2b). It is predicted that human Piezo1 and Piezo2 adopt a similar structure. A major structural difference between TRP and Piezo channels is the number of TM domains. Each TRP channel has 6 TM domains, while a single Piezo channel has at least 26 TM regions (Fig. 2b) (Saotome et al., 2018) and bioinformatic analysis of protein structure predicts that Piezo1 can have up to 40 TM domains (Coste et al., 2015). Due to the large size of Piezo channels, a local concave curvature of the plasma membrane is induced around Piezo1 (Fig. 2b); upon membrane stretch, the local Piezo curvature rearranges to the convex form of the plasma membrane and the pore-forming domain is exposed, activating the channel (Zhao et al., 2018) (Fig. 2b).

Piezo channels have been activated in vitro by a variety of mechanical stimulus including stretch by substrate deformation (Koser et al., 2016), severe stress through a shear flow (Ranade et al., 2014), localized indentation with a blunt probe (Poole et al., 2014), microchannel confinement (Hung et al., 2016) and microscopic stimulation with magnetic nanoparticles (Wu et al., 2016). Piezo1 is essential for development, a global Piezo knock-out in mice is lethal mainly through failure of vascular development (Ranade et al., 2014; Li et al., 2014), indicating the vital role mechanosensors play in cell biology and the importance of local regulation of Ca^2+^ levels. Altered Piezo1 expression is associated with several aggressive cancers including breast cancer (Yu et al., 2020), gliomas (Zhou et al., 2020; Qu et al., 2020) and squamous cell carcinoma (Hasegawa et al., 2021).

## 4 MS ion channels in cell migration – mechanisms of action

MS ion channels have been linked to several biological processes that directly affect cell migration. Ca^2+^-dependent processes that lead to actin remodelling, myosin II contractility, maintenance of persistence, the establishment of directionality, binding to and integrity of the ECM are all regulated by MS ion channels. Moreover, MS ion channels control changes in gene expression that affect important cell migration processes such as epithelial to mesenchymal transition (EMT). In this section, we analyse the role of each MS ion channel in cell migration, highlighting the similarities in the mechanisms of action between different MS ion channels, which might provide strong evidence for a unified mechanism of action. We also discuss antagonistic effects between MS ion channels, which show the complexity of MS ion channel-mediated mechanotransduction.

### 4.1 Actomyosin regulation

Actin cytoskeleton remodelling is key in the process of cell migration. Changes to the actin-based structures directly translate to cell shape modifications, and to the formation of actin-rich structures such as lamellipodia and filopodial protrusions, which are essential for mesenchymal cell movement. Actin stress fibres that connect to focal adhesions provide a link between mechanosensors and the cytoskeleton and are required for integrin-based migration (Seetharaman and Etienne-Manneville, 2020). Actomyosin contractility at the back of migrating cells and actin retrograde flow is observed in all modes of cell migration and provides the pushing force for cell movement (Yolland et al., 2019). Tight regulation of the formation and dynamics of the actin structures is essential for cell migration. One of the most prominent mechanisms of action for MS ion channels in regulating the behaviour of migrating cells is linked to actin cytoskeleton rearrangement, protrusion dynamics and interaction of MS ion channels with actin-binding proteins.

#### 4.1.1 Actin remodelling

Changes in cell shape and an increase in actin remodelling occur after TRPV4 and Piezo1 chemical activation, by 4α-PDD and Yoda1 respectively (Martin et al., 2012; Chubinskiy-Nadezhdin et al., 2019; Lee et al., 2016). The mechanism of action appears to involve direct interaction of TRPV4 and Piezo1 with actin fibres and with actin-binding proteins. TRPV4 can induce a re-arrangement of the microtubule network by physically interacting with tubulin in smooth muscle cells, leading to increased cell migration (Martin et al., 2012). In contrast, Piezo1 activation leads to increased stress fibre formation and a more stable cytoskeleton structure, which leads to decreased cell migration in fibroblasts (Chubinskiy-Nadezhdin et al., 2019). While both Piezo1 and TRPV4 have been linked to roles in regulating actin structures and modifying cell shape, their function appears to have high specificity and the overall effect on cell migration can drastically change depending on the specific actin fibres interacting with the MS ion channel. Furthermore, TRPV4 can prevent phosphorylation of the ERM actin-binding proteins (ezrin, radixin and moesin) in breast cancer cells, TRPV4 mediated decrease in ERM phosphorylation is accompanied by a switch in the mechanism of cell migration from a mesenchymal mode based on actin protrusions to amoeboid with high membrane blebbing (Lee et al., 2016).

#### 4.1.2 Regulation of the small GTPase Rac1

Actin-based protrusions can be regulated by MS ion channels by regulating the activity of the small GTPase Rac1. Immunostaining and FRET experiments in keratinocytes and endothelial cells show co-localization between TRPV4 and filamentous actin (F-actin) specifically in highly dynamic actin structures, such as filopodia and lamellipodia (Becker et al., 2009; Fiorio Pla et al., 2012; Yang et al., 2020). In GN11 cells, TRPV4 activation leads to a retraction of the lamellipodia and a decrease in migratory behaviour and cells migrate in a slow and non-directional manner (Zaninetti et al., 2011). TRPC1 can also negatively regulate protrusion dynamics, TRPC1 KD in MDCK cells leads to accelerated cell lamellipodial protrusions and cell migration (Fabian et al., 2012).

**Fig. 3:**
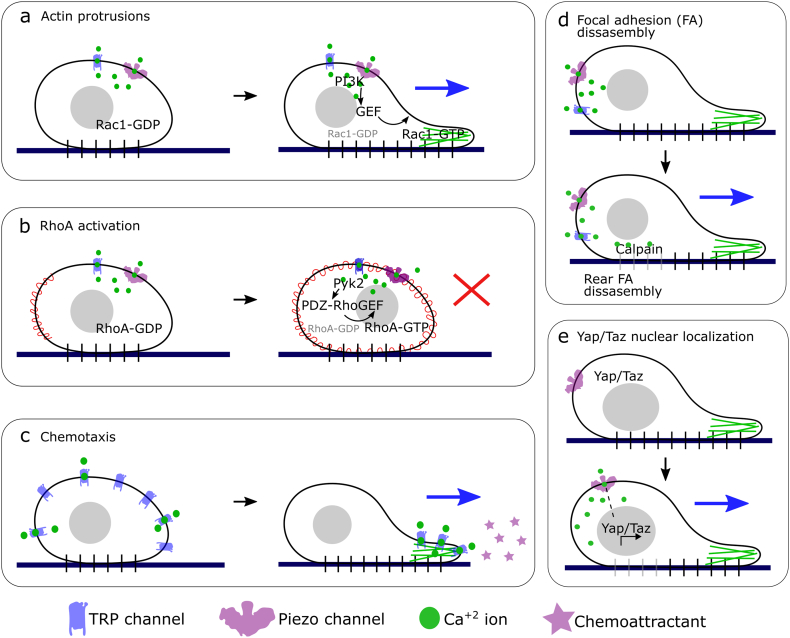
Role of MS ion channels in cell migration. **(a)** Actin protrusions. MS ion channels can regulate the extension of actin-based protrusions through PI3K signalling. Ca^2+^ binding to PI3K leads to the activation of several Rac1-GEFs, including P-Rex1 and SWAP-70, Vav1, Sos1. Rac1-GEFs mediate the transition from inactive Rac1-GDP to Rac1-GTP, leading to actin polymerization and protrusion extension. **(b)** RhoA activation. The Ca^2+^ sensitive Pyk2 kinase is activated after MS ion channel opening. Pyk2 activates PDZ-RhoGEF which mediates the transition from inactive Rho-GDP to Rho-GTP, leading to Myosin II phosphorylation. Global Myosin II contraction leads to inhibition of cell migration. **(c)** Chemotaxis. The presence of a chemoattractant agent leads to re-localization of TRPC1 and TRPC6 MS ion channels to the direction of the chemoattractant signal. Localized Ca^2+^ can regulate actin remodelling via PI3K or induce Ca^2+^ flickers at the leading edge of the cell, promoting directional cell migration. **(d)** Focal adhesion (FA) disassembly. MS ion channels regulate FA disassembly via calpain, a Ca^2+^ dependant protease that mediates FA degradation. Restricted calpain activity at the rear of the cell mediates specific FA disassembly at the back of the cell, promoting cell migration. **(e)** Yap/Taz nuclear localization. Piezo1 activation is correlated with Yap translocation from the cytoplasm to the nucleus, leading to Yap mediated gene transcription. However, the biochemical signals downstream of Piezo1 have not been identified yet. Dashed line represents unknown signalling proteins.

The mechanism of protrusion regulation appears to be linked to MS ion channel direct and indirect activation of the small GTPase Rac1. A downregulation of Piezo1 or TRPC5 correlates with the accumulation of the active form of Rac1 in gastric cancer and podocyte cells respectively (Zhang et al., 2018; Tian et al., 2010). TRPC5 is found to physically interact with Rac1 in podocyte cells and the expression of a dominant-negative form of Rac1 in TRPC5 KD cells can rescue the otherwise inhibited cell migration (Tian et al., 2010), suggesting that Rac1 acts downstream of TRPC5. TRPV4 activation in glioblastoma cells is found to promote the activation of Rac1 (Ou-Yang et al., 2018). MS ion channels could modulate Rac1 activity directly by increasing Ca^2+^ entry to the cell, as Rac1 activity has been shown to aberrantly increase by high and prolonged Ca^2+^ levels (Hayashi et al., 2018). Alternatively, Rac1 can be activated indirectly by MS ion channels through the phosphoinositide 3-kinase (PI3K) signalling pathway; TRPC6 and TRPC1 have been found to affect PI3K activity (Chaudhuri et al., 2016; Zhang et al., 2020). PI3K signalling has been linked to several biological processes including cell survival, migration and protein synthesis. Among the PI3K effectors are several Rac-GEFs (HCE et al., 2003), including P-Rex1, SWAP-70, Vav1 and Sos1 (Shinohara et al., 2002; Han et al., 1998; Das et al., 2000; Innocenti et al., 2003); which mediate the transition between the inactive GDP-bound and the active GTP-bound states of Rac1 (Fig. 3a).

MS ion channels can additionally affect PI3K through Akt, the PI3K canonical effector. Akt expression and activity has been linked to cell migration via several different mechanisms. Akt can mediate the phosphorylation of girdin, an actin-binding protein that promotes stress fibre formation and lamellipodia, this can represent an additional mechanism for MS ion channel regulation of actin structures. Akt1 signalling also enhances matrix metalloproteinase-2 (MMP2) activity in mouse mammary epithelial cells. Additionally, PI3K activation leads to the phosphorylation of the 3′-hydroxyl group of the inositol ring of phosphatidylinositol-4,5-bisphosphate (PIP_2_) to generate phosphatidylinositol-3,4,5-trisphosphate (PIP_3_). PIP_3_ is a critical lipid second messenger that recruits cytosolic proteins containing pleckstrin homology (PH) domains to the plasma membrane to promote either their activation or co-localization with other effector proteins. TRPV channels have a PIP_2_ binding domain in the C-terminal region (Fig. 2b). Recruitment of TRPV4 to pseudopodia is dependent on the activation of the PI3K/AKT pathway (Gambade et al., 2016) and the translocation of TRPC6 to the cell membrane is found to be dependent on PI3K activation to PIP_3_ (Chaudhuri et al., 2016).

The effect of MS ion channels in AKT activity has been explained by the calcium-dependent nature of AKT. TRPV4 in metastatic breast cancer was linked to cell migration by a calcium dependent regulation of AKT (Lee et al., 2017). Downregulation of Piezo1 that considerably suppressed Ca^2+^ signal increments, inhibits the phosphorylation of Akt in human prostate malignant tumour tissues, preventing Akt activation (Han et al., 2019). Additionally, silencing of TRPC1, TRPV2 and TRPV4 leads to reduced Akt gene expression (Che et al., 2016; Chung et al., 2015). However, the mechanism that links MS ion channels with changes in AKT gene expression levels is not understood. It is noteworthy that MS ion channels have been linked to changes in gene expression of additional genes, not only AKT. This will be discussed in Section 4.4.

#### 4.1.3 Regulation of small GTPase RhoA

The main forces that propel cell migration are driven by the binding of myosin II to actin filaments, specific myosin II localization at the back of the migrating cells drives the contractility to push the cells forward. Myosin II localization and activity regulation is crucial for cell migration. The tensional force of myosin II is regulated by the phosphorylation of the myosin regulatory light chain (MRLC), which is partly regulated by ROCK, an effector of the small GTPase RhoA. Regulation of RhoA activity, and therefore of myosin II, has been linked to various MS ion channels. TRPC6 forms a molecular complex with RhoA in fibroblasts and kidney podocytes (Tian et al., 2010). TRPC6-mediated Ca^2+^ influx increases RhoA activity, via the calcium dependent Pyk2, leading to ubiquitous myosin II activation within the cell thereby inhibiting cell migration (Tian et al., 2010) (Fig. 3b). RhoA activity also has a positive correlation with Piezo1 activation, total RhoA is decreased in Piezo1 knockdown gastric cancer cells (Zhang et al., 2018). In contrast, TRPV4 has the opposite effect on RhoA activity. Loss of TRPV4 leads to aberrant mechanosensitivity and a significant increase in basal Rho activity in endothelial cells (Adapala et al., 2016; Thoppil et al., 2016).

Most of the evidence suggests RhoA as a downstream effector of MS ion channels; however, the reverse has also been observed, where RhoA activity regulates the gating upstream of TRPC1. RhoA physically associates with TRPC1 to form a RhoA/TRPC1 complex. Inactivation of RhoA can reduce RhoA/TRPC1 complexes and inhibit Ca^2+^ influx, which is paralleled by an inhibition of cell migration (Chung et al., 2015), raising the question of whether TRPC1 activation requires RhoA activity. The myosin II driven forces and the cytoskeletal reorganization required for the initiation and maintenance of cell migration induce self-generated forces that feedback into the cytoskeleton leading to MS channel activation. Piezo1 can be activated by myosin II-driven intrinsic forces alone (Koser et al., 2016). This feedback loop could be the reason for the requirement of RhoA for TRPC1 activation and possibly of other MS ion channels.

### 4.2 Chemotaxis

Initiation and direction of cell migration are guided by specific mechanical and chemical cues that the cells can detect and follow. Within the chemical cues, the process of chemotaxis describes the movement of cells towards an increasing gradient of a specific chemical signal, thus establishing the directionality of cell movement. Chemotaxis is observed in migrating cells both in development and disease, notable examples include immune cells migrating towards bacterial chemoattractants during infection (Sokol and Luster, 2015), neural crest collective cell migration in the direction of SDF1 (Shellard and Mayor, 2016) and fibroblasts in response to platelet-derived growth factor (PDGF) during wound healing. (Schneider et al., 2010). The process of chemotaxis begins when a receptor detects the specific ligand, the cell polarizes actin protrusions in the direction of the increasing chemoattractant concentration and cell migration is directed towards the target tissue. Several MS ion channels have been shown to respond to gradients of specific chemoattractants (Table 1). Furthermore, chemotaxis is blocked when MS ion channels are chemically or genetically inhibited (Fabian et al., 2012; Damann et al., 2009), suggesting an essential role for MS ion channels in directional cell migration through chemotaxis.

**Table 1:** MS ion channels as chemoattractant receptors.

MS ion channel	Chemoattractant	Cell type	Reference
TRPC1	EGF	Glioblastoma	Cuddapah et al., 2013; Bomben et al., 2011
	PDGF	Glioblastoma	Lepannetier et al., 2016
	FGF-2	MDCK	Fabian et al., 2011
TRPC6	CXCR2	Neutrophils	Lindemann et al., 2013
	CXCL1	Neutrophils	Lindemann et al., 2020
	CXCL2	Neutrophils	Damann et al., 2009
TRPV2	FCS	Macrophages	Link et al., 2010
	CSF	Macrophages	Link et al., 2010
TRPV4	PAF	Neutrophils	Yin et al., 2016

In terms of a mechanism of action, the subcellular localization of MS ion channels is affected by chemoattractant presence. Immunocytochemistry of TRPC1 in the absence of PDGF shows the channel is distributed across the plasma membrane of glioblastoma cells. In contrast, when a gradient of PDGF is added, TRPC1 translocate to the protrusions at the leading edge of the cell (Lepannetier et al., 2016) (Fig. 3c). The mechanism for TRPC1 translocation was found to be dependent on PI3K mediated transport (Lepannetier et al., 2016). TRPC6 role in chemotaxis is also linked to PI3K signalling, specifically, TRPC6 can affect phosphorylation of AKT and MAPK downstream of activation via the CXCR2 receptor and lead to altered remodelling of actin fibres (Lindemann et al., 2013).

MS ion channels, as residents of the very dynamic plasma membrane, are also subject to rearrangements of the lipid bilayer. Decreased membrane fluidity reduces TRPV2 translocation to protrusions and cells cannot respond to chemoattractant signals (Gambade et al., 2016). TRPC1 channel translocation to the protrusions at the leading edge has been attributed to lipid raft proteins. TRPC1 was shown to co-localize with lipid raft proteins caveolin-1 and β-cholera toxin (Bomben et al., 2011; Huang et al., 2015). However, in vivo studies of lipid rafts have not been carried yet and there is high controversy as to whether lipid rafts are an artefact of in vitro studies. Whether TRPC1 localization in response to a chemoattractant is affected by PI3K signalling, cell membrane microdomain or other mechanisms is still not fully understood. Ca^2+^ signalling can represent a mechanism for the role of MS ion channels in chemotaxis, Ca^2+^ levels are increased at the leading edge of the cell during cell migration and local calcium changes at the site of chemotactic stimulation are essential for inducing cell polarization (Collins and Meyer, 2009). MS ion channel translocation to the leading edge of the cell could be required to induce the local Ca^2+^ increase during chemotaxis. Several questions are still open about the role of MS ion channels in chemotaxis; are MS ion channels responsible for calcium increase at the protrusions during cell migration? What are the mechanical consequences of MS ion channels localizing to the leading edge of the cells? To what extent is chemotaxis affected/influenced by mechanical stimuli? MS ion channels represent an exciting opening of possibilities joining biomechanics, calcium signalling and complex biological processes.

### 4.3 Integrins, focal adhesions and MMP activity

MS ion channels can additionally regulate cell migration by modifying the interactions of cells with the ECM. MS ion channels can regulate integrins, focal adhesions (FA) and matrix metalloproteinases (MMPs). As mentioned, the integrin/FA complex is an important cell mechanosensor. The mechanical strain received by β1 integrin and FA proteins can be transmitted to MS ion channels localized at actin protrusions and induce their activation (Matthews et al., 2010). However, MS ion channels are also known to regulate integrin disassembly. Piezo1 activity causes a switch to an integrin-free mode of cell migration in small cell lung cancer (SCLC) (McHugh et al., 2012) and decreased integrin β1 protein levels are also detected in Piezo1-knockdown cells (Yang et al., 2014). FA proteins are also affected by MS ion channel activity through a mechanism of action that appears to be dependent on the activity of calpain, a calcium-sensitive protease implicated in FA disassembly (Fig. 3d). Disrupting Ca^2+^ influx via TRPC1, Piezo2 or TRPV4 KD reduces calpain activity and consequently, larger focal adhesions are seen in TRPV4 KD HEK293 cells, T47D and U87 cells (Schaar et al., 2016; Mrkonjić et al., 2015; Pardo-Pastor et al., 2018).

MS ion channel activity can also mediate the degradation and composition of the ECM. Matrix composition can be altered by blockade or knockdown of TRPC6, which leads to decreased expression of the ECM protein fibronectin (Yang et al., 2017). Silencing of TRPV2 by small interfering RNA diminishes the expression of degrading enzymes MMP2, MMP9, and cathepsin B and decreases the formation of metastasis in PC3 prostate tumours established in mice xenografts and bladder tumour development and progression (Monet et al., 2010; Liu and Wang, 2013). Additionally, Piezo1 activation leads to an increase in the expression of multiple ECM remodelling proteins in glioblastoma cells, including TIMP1, MMP2, MMP9 (Zhou et al., 2020). Overall, the role of MS ion channel in regulating the integrity of the ECM is linked to the regulation of the expression of ECM regulating proteins, highlighting a critical role for MS ion channels in gene expression control.

### 4.4 Gene expression

The extent of the role of MS ion channels in cell migration extends beyond the cytoplasm and cytoskeleton of the cell. As mentioned in previous sections, signalling downstream of MS channels can lead to transcriptional regulation of genes that are essential for cell migration. Microarray analysis of endothelial progenitor cells showed that 13 genes had altered levels of expression following TRPC1 silencing (Kuang et al., 2012). Regulation of gene expression through MS ion channels is indirect, meaning that the mechanical stimuli have not been directly associated with changes in gene expression. Instead, gene expression alterations mediated by Piezo1 channels are driven by interaction with mechanosensitive transcription factors such as Yap/Taz (yes-associated protein/transcriptional coactivator with PDZ-binding motif) (Pathak et al., 2014) (Fig. 3e). Moreover, TRPV4 inhibition leads to softening of the ECM, which prevents nuclear translocation of Yap/Taz (Sharma et al., 2019), leading to misregulation of Yap associated genes, including Wnt/β-catenin signalling-related genes which can also be downregulated by TRPV2 and Piezo2 depletion (Kudou et al., 2019; Yang et al., 2016).

A common mechanism that is affected by altered MS ion channel activity is epithelial to mesenchymal transition (EMT), mainly via regulation of the ERK signalling pathway. TRPC1 inhibition attenuates the TGF-β1-induced EMT in gastric cancer by suppressing Ras/Raf1/ERK signal transduction (Ge et al., 2018). Pharmacological inhibition of TRPV4 channels can also repress TGFβ1 and p-ERK expression and block the EMT process in HCC cells and mouse primary epidermal keratinocytes in vitro (Sharma et al., 2019; Fang et al., 2018). In colon cancer cells, overexpression of TRPC5 causes decreased E-cadherin, and increased mesenchymal biomarker expression, thus promoting mesenchymal cell migration (Chen et al., 2017).

## 5 Conclusions and future directions

Mechanotransduction is an essential albeit poorly understood process. MS ion channels provide a substantial understanding of how mechanical forces transform to biological processes, as MS ion channels are involved in the regulation of several cell migration mechanisms via Ca^2+^ dependent processes and less understood mechanisms, like the regulation of gene expression. While the field is growing and there is substantial evidence that links MS ion channels to cell migration, there are still several questions that need to be addressed. A mechanism of action for the MS ion channel-mediated regulation of gene expression is urgently needed. While Piezo1 has been linked to the activity of the transcription factors Yap/Taz (Pathak et al., 2014), it is unknown whether Piezo1 might regulate gene expression in a Yap/Taz independent manner. Furthermore, there is still no clear mechanism of action for TRP channels in the regulation of gene expression. Additionally, MS ion channels are found localized in the membrane of different organelles, such as endoplasmic reticulum, lysosomes and other vesicles (Zhang et al., 2017; Abe and Puertollano, 2011); however, the role of MS ion channels in the cell organelles remains very poorly studied and it not known if MS ion channel intracellular signalling contributes to the regulation of cell migration.

Moreover, the mechanism for the mechanical activation of TRP channels is not well established. Identifying the mechanogating mechanism for TRP channels is a priority, as there is still controversy as to whether TRP channels are mechanosensitive. Furthermore, TRP channels are known to be activated by several chemical and mechanical signals, and it is imperative to understand how TRP channels respond to simultaneous signals within the cell. Additionally, most TRP channels have been categorized as non-mechanosensitive, yet all TRP channels are highly similar in sequence, motifs and domains; understanding what protein conformations, or what specific motifs confer mechanosensitivity to the specific MS TRP channels could provide a frame of reference for identifying completely uncharacterized MS ion channels. Finally, some MS ion channels are only beginning to be associated with either mechanotransduction or cell migration, such as TREK, P2X7 and Elkin1 (Patkunarajah et al., 2020; Sauter et al., 2016; Zhu et al., 2021). Further studies of emerging MS ion channels will be essential to consolidate their role in cell migration. Overall, the role of MS ion channels in cell migration is a rapidly growing field that provides a fascinating understanding of the regulation of complex processes such as the role of mechanics in cell biology.

## CRediT authorship contribution statement

BCC and RM are the only authors of this manuscript.
